# Redetermination of germacrone type II based on single-crystal X-ray data

**DOI:** 10.1107/S2414314624003468

**Published:** 2024-04-26

**Authors:** Florian Meurer, Michael Bodensteiner, Iliyan Kolev

**Affiliations:** a Faculty of Chemistry and Pharmacy, University of Regensburg, Universitaetsstrasse 31, 93053 Regensburg, Germany; b Faculty of Pharmacy, Department of Pharmaceutical Chemistry, Medical University ‘Prof. Dr. Paraskev Stoyanov’ – Varna, 84 "Tzar Osvoboditel" Blvd, 9000 Varna, Bulgaria; Vienna University of Technology, Austria

**Keywords:** crystal structure, germacrone, Hirshfeld atom refinement, Hirsfeld surface analysis, synthesis, extraction

## Abstract

The extraction and purification procedures, crystallization and crystal structure refinement (single-crystal X-ray data) of germacrone type II, C_15_H_22_O, are presented. The structural results are compared with a previous powder X-ray synchrotron study [Kaduk *et al.* (2022 ▸). *Powder Diffr.* **37**, 98–104], revealing significant improvements in terms of accuracy and precision. Hirshfeld atom refinement (HAR), as well as Hirshfeld surface analysis, give insight into the inter­molecular inter­actions of germacrone type II.

## Abstract 2

The crystal structure model of germacrone type II determined from single-crystal X-ray data is compared with that of a previous synchrotron X-ray powder study

## Graphical abstract

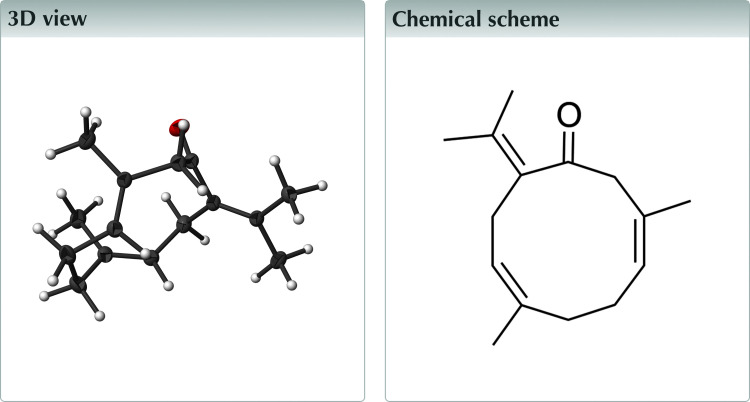

## Structure description

(3*E*,7*E*)-3,7-Dimethyl-10-propan-2-yl­idene­cyclo­deca-3,7-dien-1-one (**1**), also called germacrone, is dimorphic. The first polymorph was reported in 1999 based on single-crystal X-ray data (Clardy & Lobkovsky, 1999 ▸), and the second polymorph (germacrone type II) in 2022 based on synchrotron powder X-ray diffraction data (Kaduk *et al.*, 2022 ▸). Herein we compare the results of our single-crystal X-ray study with the mol­ecular structure refined with the Rietveld method (Kaduk *et al.*, 2022 ▸).

**Figure 1:**
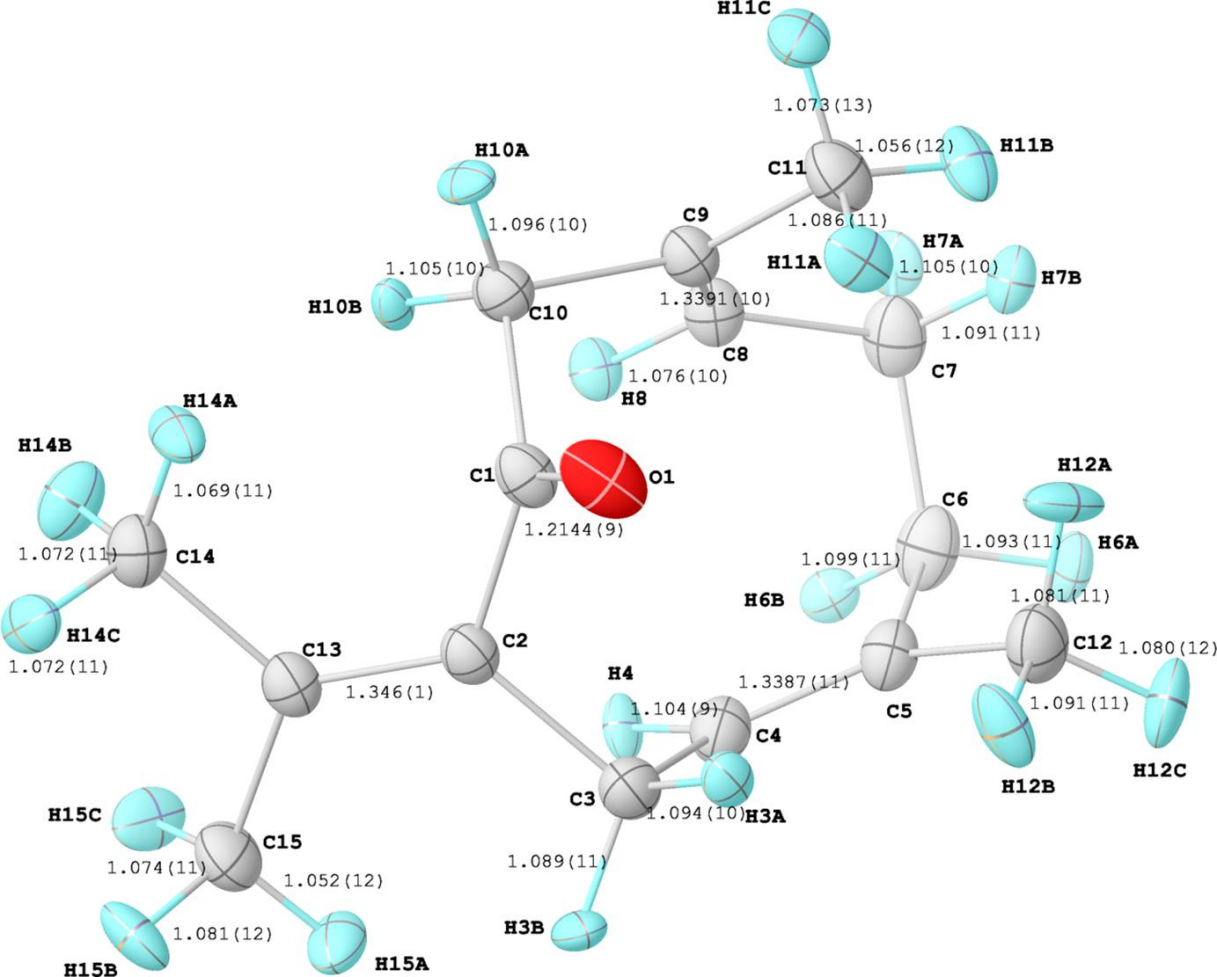
The mol­ecular structure of (**1**) with the atomic labelling scheme. Anisotropic displacement ellipsoids are drawn at the 50% probability level. Bond lengths (Å), except for C(*sp* ^3^)—C(*sp* ^3^) and C(*sp* ^2^)—C(*sp* ^2^) bonds, are indicated.

**Figure 2:**
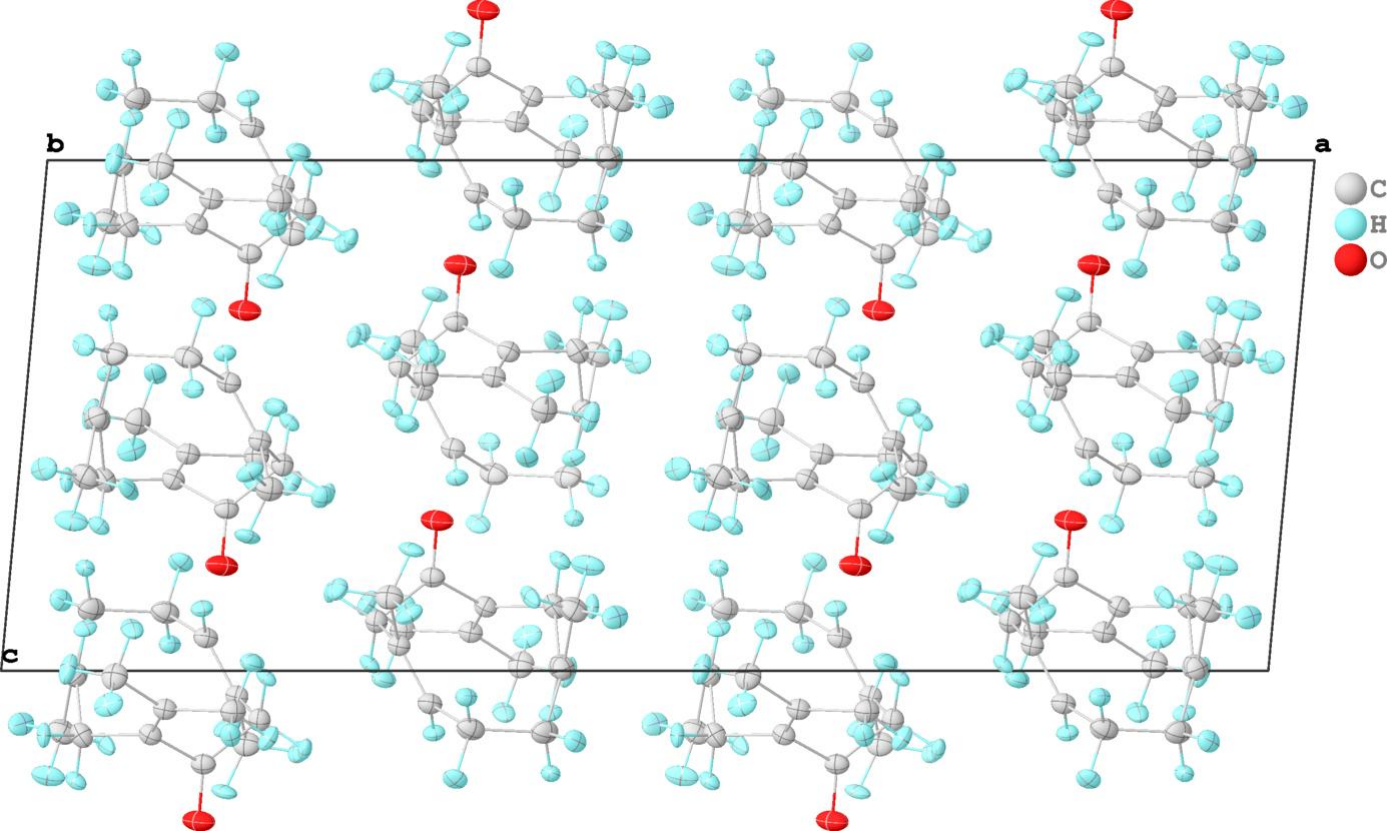
Crystal packing of (**1**) along the crystallographic *b* direction. Anisotropic displacement ellipsoids are drawn at the 50% probability level.

**Figure 3:**
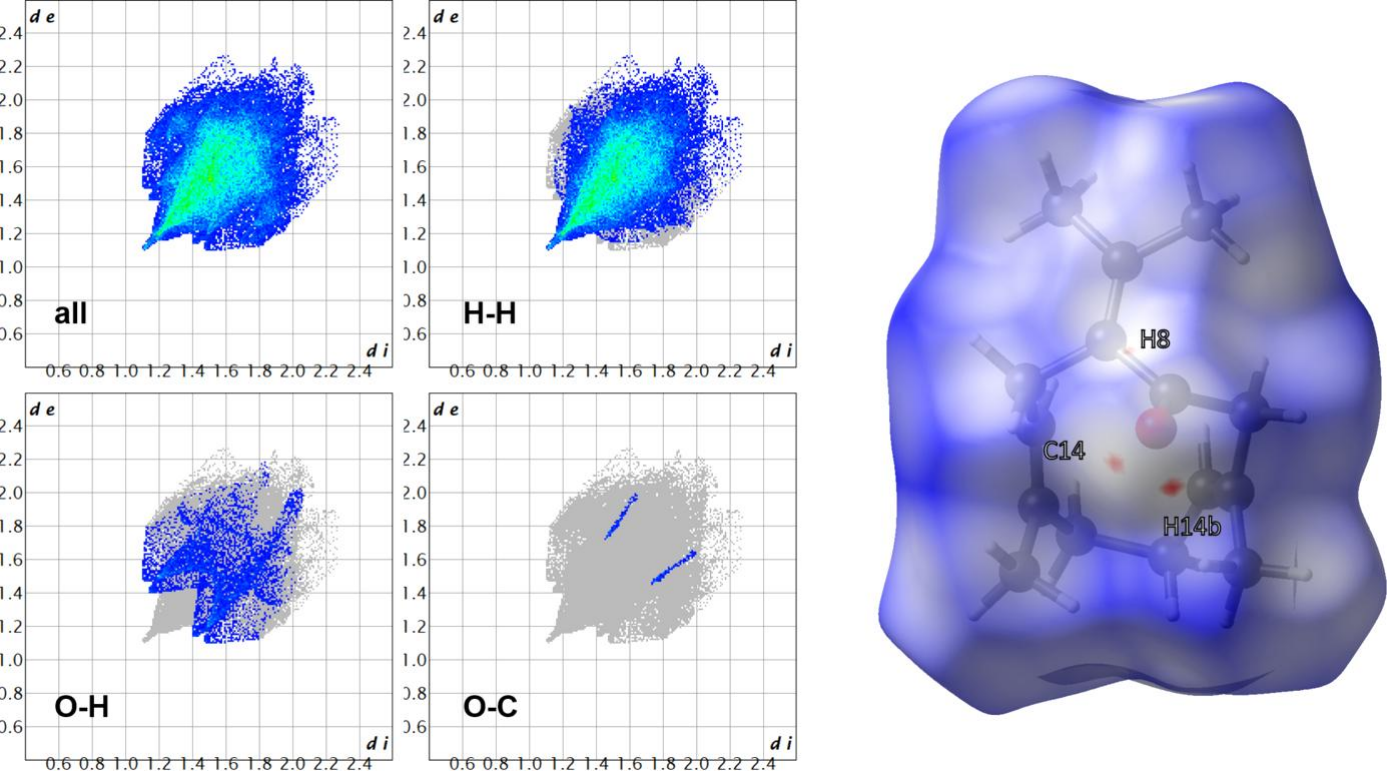
Hirshfeld fingerprint plots (left) of (**1**), showing the contacts on the Hirshfeld surface (right).

We confirm that (**1**) crystallizes in the monoclinic space group *C*2/*c*. The unit-cell volume of 2579.78 (10) Å^3^ at a temperature of 100 K is about 4% smaller than that of 2684.06 (4) Å^3^ determined at room temperature. Fig. 1 ▸ shows the mol­ecular structure of (**1**) and Fig. 2 ▸ the packing of the mol­ecules along the crystallographic *b* direction. The most prominent feature with respect to the crystal packing aspects of (**1**) is the carbonyl group (C1=O1) next to the C=CMe_2_ entity [C2=C13(C14H_3_)(C15H_3_)]. A Hirshfeld surface analysis using *CrystalExplorer* (Spackman *et al.*, 2021 ▸) reveals that the carbonyl group is responsible for the only contacts of (**1**) with its periodic environment, with distances below the sum of the van der Waals radii (Fig. 3 ▸, red contacts). Numerical details of the contacts involving H atoms below 5 Å are listed in Table 1 ▸. In comparison with the room-temperature powder study, we found longer hydrogen–acceptor (H⋯*A*) distances, *e.g.* with one of the shortest H⋯*A* contacts being 2.59 (1) Å, while it was reported at 2.473 Å by Kaduk *et al.* (2022 ▸). A possible reason for this difference may be that we refined C—H distances directly based on the single-crystal X-ray diffraction data, employing Hirshfeld Atom Refinement (HAR). It has been reported that HAR yields C—H bond lengths that are as accurate as neutron data (Woińska *et al.*, 2016 ▸), so we are confident that these distances for germacrone type II are improved compared to the previous powder study.

**Figure 4:**
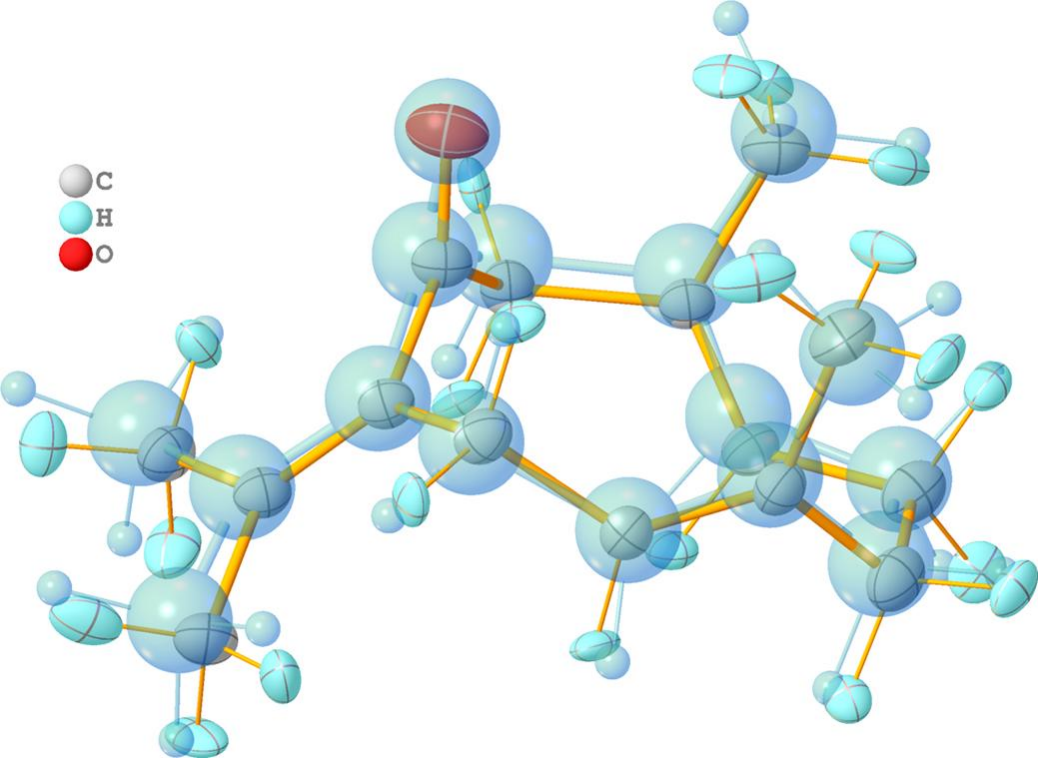
Overlayed mol­ecular structures of germacrone type II determined in this work (ellipsoids connected by orange bonds) and from previous powder data (Kaduk *et al.*, 2022 ▸; blue spheres). Ellipsoids and spheres are drawn at the 50% probability level.

In Table 2 ▸, the bond lengths between all atoms heavier than hydrogen are compared between the current single-crystal X-ray study and the previous powder study by Kaduk *et al.* (2022 ▸). The accuracy of the bond lengths differs by an entire order of magnitude and some distances differ strongly. For example, the C5—C12 bond to the methyl group of C12 is heavily underestimated [1.395 (12) Å] compared to 1.5017 (10) Å determined in the current study. The higher accuracy and precision of the current model results from the single-crystal X-ray data and the use of a successful non-spherical description of the atoms, but also from the low-temperature data. The overlap of both mol­ecular structures (Fig. 4 ▸) underlines the difference between the two structure refinements.

However, the Hirshfeld surface analysis (Fig. 3 ▸) is in close agreement with the results by Kaduk *et al.* (2022 ▸). The inter­molecular inter­action in germacrone type II is of primarily dispersion character of H⋯H contacts (81.1%), with the remainder mostly consisting of O⋯H contacts (9.5%) and O⋯C contacts (0.8%).

## Synthesis and crystallization

The essential oil (EO) from the leaves of *Geranium macrorrhizum* L. was obtained by steam distillation, using a conventional distillation vessel with a capacity of 2.5 m^3^. The target terpenoid was isolated from the resulting EO. For this purpose, approximately 1.0 g of EO was dissolved in 5.0 ml of 99% vol. ethanol. To this solution, distilled water was subsequently added dropwise until a faint opalescence appeared. The homogeneity of the latter was restored by adding 200 µl of ethanol. The resulting solution was allowed to stand in a refrigerator for several hours. The crystals formed were separated from the remaining solution and purified twice by the same methodology. Approximately 200 mg of thin acicular crystals were thus obtained.

## Refinement

Crystal data, data collection and structure refinement details are summarized in Table 3 ▸. Refinement of the initial structure solution as determined by *SHELXT* (Sheldrick, 2015 ▸) was performed using *olex2.refine* (Dolomanov *et al.*, 2009 ▸; Bourhis *et al.*, 2015 ▸). The refined structure was used as an input to perform an iterative Hirshfeld atom refinement (HAR) using *NoSpherA2* (Kleemiss *et al.*, 2021 ▸) at the R2SCAN/cc-pVDZ level of theory until convergence was reached after eight cycles. This allowed us to model all atoms, including H atoms anisotropically without any constraints or restraints on the structural model.

The final model was used to generate the input file for *CrystalExplorer* (Spackman *et al.*, 2021 ▸).

## Supplementary Material

Crystal structure: contains datablock(s) I. DOI: 10.1107/S2414314624003468/wm4211sup1.cif

Structure factors: contains datablock(s) I. DOI: 10.1107/S2414314624003468/wm4211Isup2.hkl

CCDC reference: 2349265

Additional supporting information:  crystallographic information; 3D view; checkCIF report

## Figures and Tables

**Table 1:** Hydrogen-bond geometry (Å, °)

*D*—H⋯*A*	*D*—H	H⋯*A*	*D*⋯*A*	*D*—H⋯*A*
C8—H8⋯O1^i^	1.076 (10)	2.590 (10)	3.6245 (10)	161.0 (8)
C10—H10*B*⋯O1^i^	1.105 (9)	2.695 (10)	3.7028 (10)	151.3 (7)
C14—H14*B*⋯O1^i^	1.072 (11)	2.552 (12)	3.2434 (10)	121.5 (9)
C4—H4⋯O1^i^	1.104 (9)	3.356 (10)	4.1760 (9)	132.0 (6)
C11—H11*C*⋯O1^ii^	1.073 (13)	3.177 (12)	4.1177 (11)	146.9 (9)

Symmetry codes: (i) 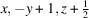 ; (ii) 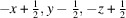 .

**Table 2:** Comparison of bond lengths (Å) determined from the current single-crystal X-ray study and from the powder study by Kaduk *et al.* (2022 ▸)

Atom	Atom	Current single-crystal X-ray study.	Previous powder study*
O1	C1	1.2144 (9)	1.212 (10)
C1	C2	1.5035 (10)	1.558 (10)
C2	C3	1.5221 (10)	1.516 (11)
C4	C3	1.5069 (11)	1.513 (12)
C5	C4	1.3387 (11)	1.314 (11)
C5	C12	1.5017 (10)	1.395 (12)
C5	C6	1.5121 (11)	1.497 (12)
C7	C6	1.5597 (12)	1.518 (15)
C1	C10	1.5292 (10)	1.514 (12)
C9	C8	1.3391 (10)	1.326 (13)
C9	C10	1.5207 (10)	1.576 (12)
C9	C11	1.5005 (10)	1.537 (13)
C8	C7	1.5002 (10)	1.484 (13)
C13	C2	1.3460 (10)	1.405 (10)
C13	C14	1.5015 (11)	1.601 (11)
C13	C15	1.5015 (11)	1.574 (11)

Note: (*) atom labels were adopted from the current single-crystal X-ray study for better comparison.

**Table 3:** Experimental details

Crystal data	
Chemical formula	C_15_H_22_O
*M*_r_	218.34
Crystal system, space group	Monoclinic, *C*2/*c*
Temperature (K)	100
*a*, *b*, *c* (Å)	25.6112 (6), 9.7565 (2), 10.3664 (2)
β (°)	95.169 (2)
*V* (Å^3^)	2579.78 (10)
*Z*	8
Radiation type	Cu *K*α
μ (mm^−1^)	0.52
Crystal size (mm)	0.35 × 0.10 × 0.01

Data collection	
Diffractometer	XtaLAB Synergy R, DW system, HyPix-Arc 150
Absorption correction	Gaussian (*CrysAlis PRO*; Rigaku OD, 2023 ▸)
*T*_min_, *T*_max_	0.601, 1.000
No. of measured, independent and observed [*I* ≥ 2u(*I*)] reflections	13789, 2579, 2244
*R*_int_	0.026
(sin θ/λ)_max_ (Å^−1^)	0.624

Refinement	
*R*[*F* ^2^ > 2σ(*F* ^2^)], *wR*(*F* ^2^), *S*	0.029, 0.077, 1.07
No. of reflections	2579
No. of parameters	343
H-atom treatment	All H-atom parameters refined
Δρ_max_, Δρ_min_ (e Å^−3^)	0.15, −0.19

Computer programs: *CrysAlis PRO* (Rigaku OD, 2023 ▸), *SHELXT* (Sheldrick, 2015 ▸), *olex2.refine* (Bourhis *et al.*, 2015 ▸) and *OLEX2* (Dolomanov *et al.*, 2009 ▸).

## full crystallographic data

### Crystal data

**Table:**

C_15_H_22_O	*F*(000) = 962.722
*M**_r_* = 218.34	*D*_x_ = 1.124 Mg m^−^^3^
Monoclinic, *C*2/*c*	Cu *K*α radiation, λ = 1.54184 Å
*a* = 25.6112 (6) Å	Cell parameters from 7327 reflections
*b* = 9.7565 (2) Å	θ = 3.5–73.5°
*c* = 10.3664 (2) Å	µ = 0.52 mm^−^^1^
β = 95.169 (2)°	*T* = 100 K
*V* = 2579.78 (10) Å^3^	Plate, colourless
*Z* = 8	0.35 × 0.10 × 0.01 mm

### Data collection

**Table:**

XtaLAB Synergy R, DW system, HyPix-Arc 150 diffractometer	2244 reflections with *I*≥ 2u(*I*)
Detector resolution: 10.0000 pixels mm^-1^	*R*_int_ = 0.026
ω scans	θ_max_ = 74.2°, θ_min_ = 3.5°
Absorption correction: gaussian (CrysAlis PRO; Rigaku OD, 2023)	*h* = −31→31
*T*_min_ = 0.601, *T*_max_ = 1.000	*k* = −12→11
13789 measured reflections	*l* = −12→7
2579 independent reflections	

### Refinement

**Table:**

Refinement on *F*^2^	0 restraints
Least-squares matrix: full	0 constraints
*R*[*F*^2^ > 2σ(*F*^2^)] = 0.029	All H-atom parameters refined
*wR*(*F*^2^) = 0.077	*w* = 1/[σ^2^(*F*_o_^2^) + (0.055*P*)^2^ + 0.0128*P*] where *P* = (*F*_o_^2^ + 2*F*_c_^2^)/3
*S* = 1.07	(Δ/σ)_max_ = 0.0002
2579 reflections	Δρ_max_ = 0.15 e Å^−^^3^
343 parameters	Δρ_min_ = −0.19 e Å^−^^3^

### Fractional atomic coordinates and isotropic or equivalent isotropic displacement parameters (Å^2^)

**Table:**

	*x*	*y*	*z*	*U*_iso_*/*U*_eq_	
O1	0.33333 (3)	0.41200 (6)	0.20570 (5)	0.03252 (17)	
C9	0.31211 (3)	0.25885 (7)	0.45034 (7)	0.02028 (17)	
C8	0.34076 (3)	0.24944 (8)	0.56458 (7)	0.02222 (18)	
C13	0.36790 (3)	0.66412 (8)	0.42455 (6)	0.02161 (18)	
C1	0.33372 (3)	0.45137 (7)	0.31692 (6)	0.02148 (17)	
C2	0.37771 (3)	0.54035 (8)	0.37471 (6)	0.02055 (17)	
C10	0.29269 (3)	0.39910 (8)	0.40343 (7)	0.02205 (18)	
C14	0.31393 (3)	0.72500 (9)	0.42034 (8)	0.02466 (18)	
C5	0.44323 (3)	0.24947 (8)	0.49580 (7)	0.02413 (18)	
C4	0.44050 (3)	0.38643 (8)	0.49131 (7)	0.02410 (18)	
C3	0.43197 (3)	0.47697 (8)	0.37352 (7)	0.02458 (18)	
C11	0.30102 (4)	0.14357 (9)	0.35603 (8)	0.02775 (19)	
C7	0.37470 (4)	0.13211 (9)	0.61428 (8)	0.02777 (19)	
C15	0.40994 (4)	0.75499 (9)	0.48836 (9)	0.02842 (19)	
C12	0.44745 (4)	0.15866 (10)	0.38024 (8)	0.0297 (2)	
C6	0.43328 (4)	0.17738 (9)	0.62037 (8)	0.0290 (2)	
H8	0.3460 (4)	0.3411 (11)	0.6219 (9)	0.041 (3)	
H10A	0.2546 (4)	0.3942 (11)	0.3461 (10)	0.038 (3)	
H10B	0.2903 (4)	0.4685 (10)	0.4870 (9)	0.034 (2)	
H14A	0.2841 (4)	0.6658 (11)	0.3672 (10)	0.048 (3)	
H14B	0.3025 (5)	0.7431 (14)	0.5158 (11)	0.061 (4)	
H14C	0.3145 (5)	0.8231 (12)	0.3739 (11)	0.049 (3)	
H4	0.4366 (4)	0.4415 (10)	0.5829 (9)	0.038 (3)	
H3A	0.4331 (4)	0.4181 (11)	0.2840 (10)	0.040 (3)	
H3B	0.4618 (4)	0.5565 (11)	0.3739 (10)	0.043 (3)	
H11A	0.3154 (5)	0.1660 (13)	0.2629 (10)	0.051 (3)	
H11B	0.3178 (5)	0.0496 (12)	0.3891 (10)	0.053 (3)	
H7A	0.3659 (5)	0.1001 (11)	0.7122 (10)	0.049 (3)	
H7B	0.3688 (4)	0.0424 (12)	0.5518 (9)	0.044 (3)	
H15A	0.4471 (5)	0.7088 (14)	0.5030 (12)	0.064 (4)	
H15B	0.4128 (6)	0.8482 (12)	0.4331 (12)	0.060 (4)	
H15C	0.3996 (5)	0.7869 (14)	0.5819 (11)	0.060 (4)	
H12A	0.4126 (5)	0.0982 (14)	0.3561 (11)	0.058 (4)	
H12B	0.4554 (6)	0.2159 (12)	0.2938 (11)	0.065 (4)	
H12C	0.4805 (5)	0.0899 (13)	0.3954 (11)	0.064 (4)	
H6A	0.4579 (5)	0.0867 (12)	0.6382 (10)	0.049 (3)	
H6B	0.4410 (4)	0.2496 (12)	0.7012 (10)	0.040 (3)	
H11C	0.2595 (5)	0.1274 (12)	0.3405 (11)	0.050 (3)	

### Atomic displacement parameters (Å^2^)

**Table:**

	*U*^11^	*U*^22^	*U*^33^	*U*^12^	*U*^13^	*U*^23^
O1	0.0470 (4)	0.0323 (3)	0.0186 (3)	−0.0112 (3)	0.0049 (2)	−0.0026 (2)
C9	0.0235 (4)	0.0174 (4)	0.0201 (3)	−0.0011 (3)	0.0027 (3)	0.0002 (3)
C8	0.0269 (4)	0.0204 (4)	0.0195 (4)	0.0022 (3)	0.0032 (3)	0.0005 (3)
C13	0.0258 (4)	0.0187 (4)	0.0205 (3)	−0.0003 (3)	0.0028 (3)	0.0001 (3)
C1	0.0274 (4)	0.0184 (3)	0.0187 (3)	−0.0019 (3)	0.0022 (3)	0.0008 (3)
C2	0.0237 (4)	0.0180 (4)	0.0203 (3)	−0.0009 (3)	0.0039 (3)	0.0002 (3)
C10	0.0220 (4)	0.0202 (4)	0.0240 (4)	−0.0005 (3)	0.0023 (3)	0.0021 (3)
C14	0.0283 (5)	0.0213 (4)	0.0242 (4)	0.0036 (3)	0.0018 (3)	0.0009 (3)
C5	0.0242 (4)	0.0231 (4)	0.0249 (4)	0.0054 (3)	0.0015 (3)	−0.0018 (3)
C4	0.0228 (4)	0.0239 (4)	0.0253 (4)	0.0028 (3)	0.0008 (3)	−0.0031 (3)
C3	0.0245 (4)	0.0218 (4)	0.0284 (4)	−0.0008 (3)	0.0070 (3)	−0.0013 (3)
C11	0.0350 (5)	0.0218 (4)	0.0264 (4)	−0.0035 (4)	0.0023 (3)	−0.0035 (3)
C7	0.0366 (5)	0.0245 (4)	0.0227 (4)	0.0066 (3)	0.0052 (3)	0.0043 (3)
C15	0.0300 (5)	0.0225 (4)	0.0323 (5)	−0.0033 (4)	0.0008 (4)	−0.0055 (3)
C12	0.0353 (5)	0.0254 (4)	0.0294 (4)	0.0043 (4)	0.0085 (4)	−0.0033 (3)
C6	0.0328 (5)	0.0296 (4)	0.0243 (4)	0.0107 (4)	0.0006 (3)	0.0028 (3)
H8	0.052 (8)	0.040 (7)	0.030 (5)	0.002 (6)	0.000 (5)	−0.006 (5)
H10A	0.021 (6)	0.045 (7)	0.048 (6)	0.001 (5)	−0.004 (5)	0.012 (5)
H10B	0.045 (7)	0.024 (6)	0.035 (5)	0.000 (5)	0.018 (5)	−0.003 (5)
H14A	0.040 (7)	0.036 (7)	0.063 (7)	−0.002 (6)	−0.018 (6)	0.000 (6)
H14B	0.065 (9)	0.083 (11)	0.036 (7)	0.021 (8)	0.010 (6)	−0.003 (6)
H14C	0.045 (8)	0.046 (8)	0.057 (7)	0.007 (6)	0.008 (6)	0.020 (6)
H4	0.056 (8)	0.027 (6)	0.031 (6)	0.010 (5)	−0.005 (5)	−0.012 (5)
H3A	0.049 (7)	0.038 (7)	0.037 (6)	−0.002 (6)	0.017 (5)	−0.005 (5)
H3B	0.021 (6)	0.042 (7)	0.067 (8)	−0.003 (5)	0.005 (5)	0.002 (6)
H11A	0.074 (10)	0.059 (9)	0.024 (6)	−0.011 (7)	0.015 (6)	−0.012 (5)
H11B	0.074 (9)	0.037 (7)	0.044 (7)	0.000 (7)	−0.018 (6)	−0.008 (6)
H7A	0.066 (9)	0.043 (7)	0.041 (7)	0.006 (6)	0.011 (6)	0.022 (5)
H7B	0.051 (8)	0.045 (7)	0.036 (6)	0.016 (6)	0.004 (5)	0.000 (6)
H15A	0.034 (8)	0.059 (9)	0.098 (10)	0.006 (7)	−0.012 (7)	−0.023 (7)
H15B	0.086 (11)	0.032 (7)	0.060 (8)	−0.021 (7)	−0.008 (7)	0.003 (6)
H15C	0.056 (9)	0.081 (10)	0.044 (7)	−0.013 (8)	0.011 (6)	−0.037 (7)
H12A	0.047 (8)	0.076 (10)	0.053 (7)	−0.037 (7)	0.023 (6)	−0.029 (7)
H12B	0.119 (13)	0.032 (7)	0.045 (7)	−0.005 (8)	0.018 (7)	0.008 (6)
H12C	0.072 (10)	0.061 (9)	0.058 (8)	0.041 (8)	0.001 (7)	−0.013 (6)
H6A	0.054 (8)	0.042 (7)	0.053 (7)	0.022 (6)	0.009 (6)	0.007 (6)
H6B	0.041 (7)	0.049 (7)	0.031 (6)	0.000 (6)	0.006 (5)	0.002 (5)
H11C	0.046 (8)	0.050 (8)	0.052 (7)	−0.007 (6)	−0.005 (6)	−0.013 (6)

### Geometric parameters (Å, º)

**Table:**

O1—C1	1.2144 (9)	C5—C6	1.5121 (11)
C9—C8	1.3391 (10)	C4—C3	1.5069 (11)
C9—C10	1.5207 (10)	C4—H4	1.104 (9)
C9—C11	1.5005 (10)	C3—H3A	1.094 (10)
C8—C7	1.5002 (10)	C3—H3B	1.089 (11)
C8—H8	1.076 (10)	C11—H11A	1.086 (11)
C13—C2	1.3460 (10)	C11—H11B	1.056 (12)
C13—C14	1.5015 (11)	C11—H11C	1.073 (13)
C13—C15	1.5015 (11)	C7—C6	1.5597 (12)
C1—C2	1.5035 (10)	C7—H7A	1.105 (10)
C1—C10	1.5292 (10)	C7—H7B	1.091 (11)
C2—C3	1.5221 (10)	C15—H15A	1.052 (12)
C10—H10A	1.096 (10)	C15—H15B	1.081 (12)
C10—H10B	1.105 (9)	C15—H15C	1.074 (11)
C14—H14A	1.069 (11)	C12—H12A	1.081 (11)
C14—H14B	1.072 (11)	C12—H12B	1.091 (11)
C14—H14C	1.072 (11)	C12—H12C	1.080 (12)
C5—C4	1.3387 (11)	C6—H6A	1.093 (11)
C5—C12	1.5017 (10)	C6—H6B	1.099 (11)
			
C10—C9—C8	118.81 (7)	H3A—C3—C4	111.5 (6)
C11—C9—C8	125.55 (7)	H3B—C3—C2	110.6 (6)
C11—C9—C10	115.40 (6)	H3B—C3—C4	111.4 (6)
C7—C8—C9	127.62 (7)	H3B—C3—H3A	107.7 (8)
H8—C8—C9	117.5 (5)	H11A—C11—C9	111.7 (6)
H8—C8—C7	113.9 (5)	H11B—C11—C9	113.1 (5)
C14—C13—C2	123.16 (7)	H11B—C11—H11A	107.7 (10)
C15—C13—C2	123.20 (7)	H11C—C11—C9	109.5 (6)
C15—C13—C14	113.64 (7)	H11C—C11—H11A	107.8 (9)
C2—C1—O1	120.18 (7)	H11C—C11—H11B	106.8 (9)
C10—C1—O1	120.26 (7)	C6—C7—C8	108.86 (7)
C10—C1—C2	119.26 (6)	H7A—C7—C8	111.8 (6)
C1—C2—C13	120.78 (7)	H7A—C7—C6	108.6 (6)
C3—C2—C13	124.74 (7)	H7B—C7—C8	111.3 (5)
C3—C2—C1	114.46 (6)	H7B—C7—C6	109.4 (6)
C1—C10—C9	105.39 (6)	H7B—C7—H7A	106.8 (8)
H10A—C10—C9	112.6 (6)	H15A—C15—C13	114.5 (7)
H10A—C10—C1	108.7 (5)	H15B—C15—C13	110.1 (7)
H10B—C10—C9	109.9 (5)	H15B—C15—H15A	109.4 (11)
H10B—C10—C1	110.1 (5)	H15C—C15—C13	110.1 (7)
H10B—C10—H10A	110.1 (8)	H15C—C15—H15A	106.7 (9)
H14A—C14—C13	114.5 (6)	H15C—C15—H15B	105.6 (10)
H14B—C14—C13	111.5 (7)	H12A—C12—C5	112.9 (6)
H14B—C14—H14A	108.8 (10)	H12B—C12—C5	112.7 (6)
H14C—C14—C13	108.4 (6)	H12B—C12—H12A	107.5 (9)
H14C—C14—H14A	106.8 (8)	H12C—C12—C5	111.2 (6)
H14C—C14—H14B	106.4 (9)	H12C—C12—H12A	108.4 (11)
C12—C5—C4	124.64 (7)	H12C—C12—H12B	103.6 (10)
C6—C5—C4	118.79 (7)	C7—C6—C5	109.43 (6)
C6—C5—C12	115.89 (7)	H6A—C6—C5	112.5 (6)
C3—C4—C5	128.07 (7)	H6A—C6—C7	108.4 (6)
H4—C4—C5	117.7 (5)	H6B—C6—C5	108.7 (6)
H4—C4—C3	113.2 (5)	H6B—C6—C7	108.6 (6)
C4—C3—C2	107.30 (6)	H6B—C6—H6A	109.2 (8)
H3A—C3—C2	108.4 (6)		
			
O1—C1—C2—C13	123.05 (8)	C8—C7—C6—C5	−46.11 (7)
O1—C1—C2—C3	−58.17 (8)	C13—C2—C1—C10	−63.20 (8)
O1—C1—C10—C9	80.30 (8)	C13—C2—C3—C4	94.67 (8)
C9—C8—C7—C6	110.92 (9)	C1—C2—C3—C4	−84.06 (6)
C9—C10—C1—C2	−93.45 (6)	C2—C3—C4—C5	110.80 (7)

### Hydrogen-bond geometry (Å, º)

**Table:**

*D*—H···*A*	*D*—H	H···*A*	*D*···*A*	*D*—H···*A*
C8—H8···O1^i^	1.076 (10)	2.590 (10)	3.6245 (10)	161.0 (8)
C10—H10*B*···O1^i^	1.105 (9)	2.695 (10)	3.7028 (10)	151.3 (7)
C14—H14*B*···O1^i^	1.072 (11)	2.552 (12)	3.2434 (10)	121.5 (9)
C4—H4···O1^i^	1.104 (9)	3.356 (10)	4.1760 (9)	132.0 (6)
C11—H11*C*···O1^ii^	1.073 (13)	3.177 (12)	4.1177 (11)	146.9 (9)

Symmetry codes: (i) *x*, −*y*+1, *z*+1/2; (ii) −*x*+1/2, *y*−1/2, −*z*+1/2.
